# Bottom Up Ethics - Neuroenhancement in Education and Employment

**DOI:** 10.1007/s12152-018-9366-7

**Published:** 2018-05-01

**Authors:** Imre Bard, George Gaskell, Agnes Allansdottir, Rui Vieira da Cunha, Peter Eduard, Juergen Hampel, Elisabeth Hildt, Christian Hofmaier, Nicole Kronberger, Sheena Laursen, Anna Meijknecht, Salvör Nordal, Alexandre Quintanilha, Gema Revuelta, Núria Saladié, Judit Sándor, Júlio Borlido Santos, Simone Seyringer, Ilina Singh, Han Somsen, Winnie Toonders, Helge Torgersen, Vincent Torre, Márton Varju, Hub Zwart

**Affiliations:** 1 Department of Methodology, London School of Economics, London, UK; 2 Toscana Life Sciences Foundation, Siena, Italy; 3 Institute of Molecular and Cellulaor Biology, Porto, Portugal; 4 Experimentarium, Science Communication Centre, Copenhagen, Denmark; 5 Center for Interdisciplinary Risk and Innovation Studies, Stuttgart University, Stuttgart, Germany; 6 Center for the Study of Ethics in the Professions, Illinois Institute of Technology, Chicago, IL USA; 7 Department of Social and Economic Psychology, Johannes Kepler University, Linz, Austria; 8 Tilburg Institute for Law, Technology, and Society, Tilburg University, Tilburg, The Netherlands; 9 Centre for Ethics University of Iceland, Reykjavik, Iceland; 10 Centre on Science, Communication and Society Universitat Pompeu Fabra, Barcelona, Spain; 11 The Center for Ethics and Law in Biomedicine Central European University, Budapest, Hungary; 12 Department of Psychiatry and Oxford Uehiro Centre University of Oxford, Oxford, UK; 13 Institute for Science, Innovation and Society, Radboud University Nijmegen, Nijmegen, The Netherlands; 14 Institute of Technology Assessment Austrian Academy of Sciences, Vienna, Austria; 15 Centre for Neurobiology, International School for Advanced Studies, Trieste, Italy

**Keywords:** Neuroenhancement, Social values, Empirical ethics

## Abstract

Neuroenhancement involves the use of neurotechnologies to improve cognitive, affective or behavioural functioning, where these are not judged to be clinically impaired. Questions about enhancement have become one of the key topics of neuroethics over the past decade. The current study draws on in-depth public engagement activities in ten European countries giving a bottom-up perspective on the ethics and desirability of enhancement. This informed the design of an online contrastive vignette experiment that was administered to representative samples of 1000 respondents in the ten countries and the United States. The experiment investigated how the gender of the protagonist, his or her level of performance, the efficacy of the enhancer and the mode of enhancement affected support for neuroenhancement in both educational and employment contexts. Of these, higher efficacy and lower performance were found to increase willingness to support enhancement. A series of commonly articulated claims about the individual and societal dimensions of neuroenhancement were derived from the public engagement activities. Underlying these claims, multivariate analysis identified two social values. The Societal/Protective highlights counter normative consequences and opposes the use enhancers. The Individual/Proactionary highlights opportunities and supports use. For most respondents these values are not mutually exclusive. This suggests that for many neuroenhancement is viewed simultaneously as a source of both promise and concern.

**Electronic supplementary material:**

The online version of this article (10.1007/s12152-018-9366-7) contains supplementary material, which is available to authorized users.

## Introduction

Neuroenhancement is a controversial area of science and technology that has the potential to influence fundamental aspects of our mental and behavioural functioning. It involves the use of neurotechnologies to improve cognitive, affective or behavioural functioning, where these are not judged to be clinically impaired [1]. There are a range of technologies that might result in neuroenhancing effects, such as pharmaceuticals, brain-computer interfaces, gene editing and brain stimulation. To date, much of the neuroethics literature has focused on the off-label use of pharmaceuticals intended for the treatment of medical conditions, such as attention deficit hyperactivity disorder (ADHD) or narcolepsy [2], and more recently, on transcranial electrical brain stimulation [3–5]. While surveys suggest that some students, academics and medical professionals use prescription substances to enhance their cognitive performance [2, 6–8, 9], the precise extent of this practice is difficult to ascertain. For example, estimates of student use range from 5%–35% depending on the definition of enhancement and the method of assessment [10]. Questions around enhancement have become one of the key challenges for neuroethics and over the past decade the topic has received considerable attention in both academia and in popular culture [11, 12]. A number of national and international reports have addressed the social, legal and ethical aspects of neuroenhancement. The reports call for more research into the effects and side effects of available methods and express varying degrees of scepticism about the prospect of meaningfully improving upon normal capacities ([13]; BMA [14, 15]; Comitato Nazionale per le Bioetica [16]; NCOB [17]; PCOB [18]).

Several researchers have highlighted the importance of understanding public attitudes towards neuroenhancement and of involving a wide range of stakeholders in deliberations about the governance of the field [19, 20].

An influential report on human enhancement by the European Parliament’s Science and Technology Options Assessment agency was among the first to call for the establishment of public fora, where citizens could engage in dialogue about the opportunities and challenges of enhancement, in order to work towards a normative framework for the governance of enhancement technologies [15].

In recent years a body of empirical studies about public views in relation to enhancement has emerged. In 2004 the European Social Survey asked respondents if they approved of ‘the use of medicines to improve the memory of healthy people’. Of the ten countries featured later in this paper, on average 53% of the respondents in national samples approved or strongly approved, while 23% disapproved [49]. A Eurobarometer in 2010 found that 58% thought that ‘brain and cognitive enhancement’ would have a ‘positive effect’ on ‘our way of life over the next 20 years’ with 12% saying that it would have a ‘negative effect’ [21]. Overall, the surveys show considerable interest in enhancement and at most one in five respondents expressing scepticism.

Findings from smaller scale and in-depth studies about the ethics of enhancement reveal greater scepticism and show that the public’s views are broadly similar to those discussed in the academic literature, namely, concerns about the safety of enhancers and their potential for coercion and inequity are paramount [22]. Drawing on experimental methods to study university students’ willingness to use cognitive enhancing drugs, Sattler and his team found evidence of a pragmatic approach to decision-making [23], where perceptions of acceptability were influenced by a variety of situational factors including peer pressure, the user’s performance level in the relevant domain, the safety and efficacy of the enhancer and the person’s characteristics, such as their moral evaluation of enhancement, prior use, and study habits and motivation [24, 25]. Substances with severe side effects elicited lower willingness to use than those with moderate or slight side-effects, and willingness was significantly higher when the hypothetical student depicted in the experiment was a low performer, compared to average or high performers [24]. It is notable that two out of three respondents in Sattler et al.’s sample said they would not use cognition enhancing drugs under any circumstances.

While Sattler’s factorial surveys focused on the university context to investigate the determinants of willingness to use enhancers, a series of contrastive vignette experiments by Fitz et al. [26] studied public attitudes to the most commonly discussed ethical issues around neuroenhancement; safety, pressure to use, fairness and authenticity. They found that respondents were significantly more likely to approve of neuroenhancement and accept its risks if the aim was restorative rather than enhancing, irrespective of whether the method was pharmacological or neuro-stimulation. They found evidence for the hypothesis that attitudes towards the risks and benefits of enhancement differ according to the perceived prosocial nature of the subject’s occupation who engages in the enhancement practice. The study also showed that an enhancer that supports greater effort is preferred to an enhancer that replaces effort. Irrespective of the technology in question, respondents reported a high likelihood of using enhancers in the workplace. Furthermore, in terms of fairness the study showed that the public embraces meritocratic views and cherishes hard work [26]. A subsequent study investigated public attitudes to restorative and enhancing uses of pharmaceuticals. Across twelve cognitive, affective and social domains it found that respondents were significantly more comfortable with interventions towards the norm (restoration) than with interventions above the norm (enhancement) [27]. At the same time, stakeholder studies reveal a great deal of ambivalence and the presence of conflicting ethical principles when individuals consider the acceptability of enhancement [28].

Situated within experimental neuroethics the present study was designed to provide further empirical input into discussions about the contours of a normative framework for neuroenhancement. In line with the Forlini and Hall [28] call that research should assess the morally significant values that motivate public attitudes, the study includes a focus on values and the ways in which these influence the pragmatic approach to decision-making identified in earlier studies. To our knowledge, this is the largest investigation of public attitudes to neuroenhancement to date.

## Methods

The study reported here was part of a 39-month European project called Neuroenhancement – Responsible Research and Innovation (NERRI), conducted in ten EU Member States. The project was designed to facilitate public dialogue about the ethics and desirability of neuroenhancement (referred to hereafter as NE). The project ran more than sixty public engagement events in the form of focus groups, workshops, debates, exhibitions, a hackathon and science cafes on the subject of NE. The events involved scientists, ethicists, teachers, students, military officers, medical professionals, entrepreneurs and members of the public. The events ranged from 8-person focus groups to 500 or more attending exhibitions or other live events, and from hour-long group discussions to workshops and activities spanning several days. The outcome of these events was a qualitative appreciation of issues of interest and concern about NE [29]. These insights served as the basis for developing an online survey, in which the aim was to gain a more robust understanding of the public’s views about neuroenhancement in the participating EU countries. The United States of America was included in the survey when a project participant moved there.

The survey comprised two main sections:

1. two experiments focused on the public’s views about neuroenhancement in an educational and employment context;
2. a series of broader attitude questions related to neuroenhancement;

The experiments employed the contrastive vignette technique [30], which is emerging as a powerful method for experimental neuroethics research [26], combining the causal analysis of experimentation with the large sample sizes typical of survey research. Broadly speaking, a vignette is a short account of a situation in which a protagonist with certain characteristics faces a dilemma and choses one of two available courses of action. In its simplest form the contrastive techniques involve presenting minimally different versions of a single vignette to respondents and asking all participants the same set of questions. The minimal contrasts allow experimenters to investigate how a single factor influences people’s responses.

An advantage of using vignettes for our study is that only a minority of people in our public consultation events had heard about NE. As such, asking respondents for a judgement on NE without providing them with some information about the topic would not have yielded meaningful results. With vignettes, it is possible to give an accessible and non-technical account of NE, embedding the technology in contexts familiar to respondents.

### Experiment 1 & 2 – Education and Employment

It has been established in the literature that therapeutic interventions on the brain are generally not seen as problematic by the public. However, use by non-clinical groups in certain life world situations, such as education or work, appears more contested. Such use has been among the foci of the neuroethics literature [31, 32], and was often raised by members of the public during our engagement activities, leading to lengthy discussions of the possible personal and societal benefits and risks of NE. Following Cohen’s [33] typology of enhancement technologies, the situations depicted in the education and employment experiments broadly fall into the same category. They describe reversible, non-genetic, biological enhancements that individuals choose for themselves. The enhancement targets the upper bounds of abilities that people already possess. It is somewhat debatable whether the enhancements described in the life world contexts of education and employment should be considered positional (relative to others) or absolute (of intrinsic value) goods [34].

Respondents were presented with two vignettes, one featuring NE in education, the other NE in employment. The order of presentation of the two contexts was randomised. The experiments were designed to assess whether the type of enhancing technology, its efficacy, the gender of the user, and the user’s current level of performance shape attitudes to NE.

*Efficacy* has been shown to be an important factor modulating attitudes towards enhancement [24]. It was also a key question in the qualitative consultation exercises, where a recurrent issue raised by the participants was how well NE actually works. This suggests that both pragmatic and moral assessments may be predicated on such basic facts about the interventions. We hypothesized that a smaller (10%) improvement compared to a larger (50%) improvement would elicit different responses, with higher efficacy making NE more acceptable, because more significant benefits might outweigh perceived risks and other considerations. In relation to the *performance* of the protagonist earlier studies established that lower performance correlates with higher levels of support [24]. We hypothesise that, with the prospect of a serious loss as a result of failing an examination or losing one’s job, there would be greater propensity to accept the possible risks of enhancement [35].

Furthermore, a scenario where a protagonist is acting to avert such a loss might be perceived as a quasi-therapeutic intervention [27], and thus seen as more permissible. In our public consultations, discussions often revolved around ‘pills’ and ‘electrical brain stimulation’ as two of the currently available methods of NE. The public can draw on a great degree of life-world experience in relation to pills, and a significant proportion of the neuroethics discussion has also focused on this type of NE. Electrical brain stimulation devices are more recent but their neuroenhancing effects [4], commercial availability, and uptake in the DIY community [36] have sparked debates and proposals about the regulation of such cognition enhancing devices [37]. We sought to investigate whether contrasting these two *technologies,* the more familiar, pharmacological intervention with the less familiar, technological one, would influence attitudes. We also investigate whether the *gender* of the person engaging in NE would affect responses. The analysis of experimental effects is subsequently complemented by the inclusion of sociodemographic characteristics to assess whether these correlate with certain attitudes.

Each factor had two levels; gender (male/female); type of enhancer (pill/device); enhancer efficacy (10% or 50% improvement), and current performance (good/failing). The 16 vignettes each present a short story in which the protagonist confronts a problem, which leads to a decision to use NE. Having read the vignette, respondents answer the same set of questions, capturing the outcome variables of interest. Respondents were not aware of the other conditions. The hypotheses relating to the four factors are tested with different versions of the vignette. As respondents see only one vignette this acts against the ‘good respondent’ effect, the desire to conform to mainstream expectations on moral issues [38].

Communicating possible risks as well as benefits was deemed important to make the scenarios more realistic and life-like. For employment, the risk was described as ‘occasional insomnia’, similar to Fitz et al.’s (2013), and Sattler’s studies [24]. For education, the risk was described as ‘some people get a headache after the effect wears off’. Box 1 shows the different versions of the education and employment vignettes.

Box 1 Education and Employment Vignettes – English language versions; first names were adapted to local equivalents

**Table:** 

Employment: (GENDER Paul/Jack/Emily/Sarah) is in his/her mid-30s and works full-time at a big company. Recently, his boss told him that last year his work successfully (PERFORMANCE met / failed) to meet the company’s expectations. Paul is determined to get a promotion. This raised Paul’s fears that he could lose his job. He recently came across the idea of using (TYPE OF ENHANCER a pill / device) that promises to somewhat / substantially increase a healthy person’s concentration and memory, by about (ENHANCER EFFICACY 10% / 50%). (for device only: It delivers tiny electrical currents to stimulate certain areas of the brain using small sticky pads attached to the outside of the head for about 15 min.) In some people, the pill / device can cause occasional insomnia, but there are no known long-term side effects. He decides to give it a try. Education: (GENDER Emily/Jack/Sarah/Paul) is in his/her early twenties and studying full-time at university. His/Her results so far have been (PERFORMANCE good / below average). S/he is currently preparing for his/her examinations. While s/he feels overwhelmed with how much work s/he has to do, she is aiming for the top grade. / She feels overwhelmed with how much work she has to do and fears that she may fail the exam. Recently s/he heard about a (TYPE OF ENHANCER pill / device), which promises a (ENHANCER EFFICACY small / significant improvement in the speed of learning in healthy people, but still about 10% / by about 50%). (for device only: It delivers tiny electrical currents to stimulate certain areas of the brain using small sticky pads attached to the outside of the head for about 15 min.) Some people get a headache after the effect wears off, but there are no known long-term side effects. She decides to give it a try.	

Respondents were randomly allocated a vignette from both contexts and were asked a number of questions after each presentation. Here we report on one key outcome measure:

- In (name of protagonist)‘s shoes, would you make the same choice?

We will refer to the outcome of this question as the respondent’s ‘willingness to use NE’. The full set of questions and results are available upon request. Responses were recorded on an 11-point scale, ranging from −5 to +5. We assumed that respondents could imagine themselves in the same predicament and to be able to decide whether they would support the protagonist’s choice or not. We also assumed that this question, inviting a participant script [39], would induce a more active involvement in the situation than a question asking about the extent to which they approve or support the choice or approve/disapprove of NE in general – a non-participant script.

### Claims about Neuroenhancement

After experiments 1 & 2 respondents were asked to read the following definition of neuroenhancement, which was intended to provide a neutral account of NE introducing some key ideas without influencing respondents to lean either way in their assessment.

*Scientists are learning more about how our brains work – how we remember, how we think, how we feel and how we perceive the world. This research is driven by the desire to understand the brain and to find treatments for conditions like Alzheimer's, Parkinson's, stroke, and depression. It is hoped that this work will result in new ways of intervening in brain functions to improve the mental abilities and sensory capacities of patients. At the same time, such research might also bring about ways of enhancing the capacities of “healthy” people as well (for example: improve concentration or increase memory). This is called neuro-enhancement. The stories you read on the previous pages are just two examples of many possible situations. Some are optimistic about neuro-enhancement and think that we will be able to improve our abilities. Others are doubtful because the brain is very complex and so little is known about how it functions.*

In the course of the public engagement activities conducted throughout Europe (the United States was not involved in this part of the project), researchers met regularly to discuss their experiences, insights and the issues raised by the participants. A number of recurring themes included the personal and social consequences of NE, therapy and the duty of care, the use of technology to aid human achievement, the future of humanity, potential misuse and regulation. From these themes, the research group identified fourteen commonly mentioned claims about neuroenhancement and the use of technological interventions in human achievement. These claims may be seen as the results of a bottom-up approach to identifying moral and other considerations thought to be of relevance by the public.

**Table:** Box 2 Claims about neuroenhancement

In the survey, the following instructions were presented. “Here are some views that people have expressed about neuroenhancement of healthy individuals, and its wider implications for society. Please read the statements below and show how much you agree or disagree with them, using the scale provided (−5 to +5). 1. People should be content with their talents and abilities and not use artificial means to improve their performance 2. It is an expression of human nature to try to overcome the limitations of our body and mind 3. People’s achievements should come from their own effort and not from pills and devices 4. I can imagine neuro-enhancement opening up fascinating new opportunities 5. Some people will use neuro-enhancers to cope with increasing demands in life 6. As life gets more pressured, neuro-enhancement may be the only way out 7. It is essential that public authorities oversee and control neuro-enhancement 8. Only people with a medical problem should have access to neuro-enhancement 9. People need to be protected from pressures to use neuro-enhancers 10. If a neuro-enhancer is safe, it should be available as a consumer product 11. Neuro-enhancement should never be used on children 12. Neuro-enhancement should be available to all those who might want it 13. Neuro-enhancement will increase competition between people 14. Neuro-enhancement will threaten social cohesion	

Responses were recorded on an 11-point scale, from *Strongly disagree* (−5) to *Strongly agree* (+5).

### Design and Respondents

The survey was developed using the Qualtrics web platform. The vignettes and the accompanying questionnaire were designed by the NERRI research group. Translation from English into the national languages was undertaken by members of the research group. Assiduous attention was paid to ensuring comparability of meaning of words and phrases. The vignettes underwent qualitative piloting in focus groups conducted by the Austrian team. Subsequently, the draft survey was piloted on 200 respondents in the UK. Field work was conducted between January and February 2016 in Austria, Denmark, Germany, Hungary, Italy, the Netherlands, Portugal, Spain, UK and the USA by the commercial company Respondi, which coordinates double opt-in access panels of respondents for online surveys. In Iceland, the fieldwork was carried out by Gallup, using the same quotas. In each country quota samples of persons 18 years and above, approximating the national profile of age groups, gender and level of education (tertiary or not) were selected, *N* = 11,716. The questionnaire included two trap questions to automatically disqualify respondents speeding through the survey; some 400 per country on average. Demographic information was collected at the beginning.

In the survey, the experimental vignettes were the first block of questions, followed by respondents’ level of agreement with claims about NE. The rationale for this ordering was to ensure that the effects of the experimental treatments (gender, technology, efficacy and performance) would not be influenced by the substantive content of the claims about NE. In other words, the claims might introduce respondents to arguments about NE that they had not previously encountered and these arguments might set the context for responding to the vignettes. Were this to have occurred it would not be possible to claim that changes in responses to the vignettes could be confidently attributed solely to the experimental treatments. However, with this ordering of the questions, it is possible that the experimental treatments created a context effect for responses to the claims, a possibility that we investigate.

In reporting the results of the survey, we reverse the order as the claims about NE provide informative descriptive information on respondents’ assessment of arguments for and against NE.

## Results and Discussion

### Claims about Neuroenhancement

As described above, this section of the survey included 14 claims derived from a series of public engagement activities. We report descriptive analyses of the level of agreement to these claims and then turn to multivariate methods to explore, whether there are any latent variables underlying the responses.

Table 1 shows that there is strong agreement among respondents that neuroenhancement should not be used on children. There is also strong agreement that NE should be controlled by public authorities, that people’s achievement should come from their own efforts instead of technological aids, and that people need to be protected from pressures to use enhancers. This suggests that NE is viewed as a technology with significant potential for causing harm and leading to undesirable outcomes, so strong red lines need to be drawn and mechanisms of control put in place. However, there are also indications that the desire to overcome our limitations is an integral part of being human, hinting at a view of human nature as malleable and open to improvement. While this position is strongly held by around 50% of respondents, the same proportion indicated that people should be content with their talents and abilities and not use artificial means to improve performance. Together with the statement about the importance of people’s achievement coming from effort, this may be interpreted in multiple ways. Perhaps the public is sensitive to distinctions between different ways of pushing the boundaries of what humans can accomplish. While overall it may appear desirable to surpass our mental and physical limitations, certain means of doing so, such as pills and devices, are still unacceptable. It seems that the value of effort is paramount, and a large proportion of respondents hold the view that the ‘natural’ distribution and level of talents and abilities should not be tampered with technologically. The boundaries of where and how the human pursuit of improvement becomes undesirable warrants further investigation. Recent evidence suggests that interventions requiring the activity and agency of the subject are seen more favourably than those that involve a pharmaceutical quick fix [40]. Half of the respondents strongly agree that some people will use enhancers to cope with increasing demands and that NE is likely to increase competition among people. On average, responses are slightly above the mid-point of the scale on whether NE will threaten social cohesion, whether NE will open fascinating new opportunities and on questions concerning the group of people who should have access to NE. The average response to whether NE should be available to all is just below the mid-point. Finally, average responses are just below the mid-point, with only 20.5% of respondents agreeing that NE might be the only solution to increasing pressures in life.

**Table 1:** Mean, standard deviation and percentage in agreement with claims

Claim	Mean (SD)	% Agree*
Neuro-enhancement should never be used on children	8.0 (2.81)	76
It is essential that public authorities oversee and control neuro-enhancement	7.7 (2.52)	74
People’s achievements should come from their own effort and not from pills and devices	7.5 (2.57)	69
People need to be protected from pressures to use neuro-enhancers	7.3 (2.51)	66
It is an expression of human nature to try to overcome the limitations of our body and mind	7.2 (2.18)	68
Some people will use neuro-enhancers to cope with increasing demands in life	7.2 (2.11)	71
People should be content with their talents and abilities and not use artificial means to improve their performance	6.7 (2.85)	57
Neuro-enhancement will increase competition between people	6.6 (2.64)	58
Neuro-enhancement will threaten social cohesion	6.0 (2.76)	45
I can imagine neuro-enhancement opening up fascinating new opportunities	5.9 (2.64)	46
Only people with a medical problem should have access to neuro-enhancement	5.7 (2.97)	45
If a neuro-enhancer is safe, it should be available as a consumer product	5.6 (3.00)	43
Neuro-enhancement should be available to all those who might want it	4.8 (3.22)	34
As life gets more pressured, neuro-enhancement may be the only way out	3.8 (2.83)	21

*0, 1, 2, 3: disagree; 4, 5, 6: neutral; 7, 8, 9, 10: agree

Next, the claims are investigated with multivariate methods. Using principal components analysis, two components with eigenvalues larger than 1 were extracted, which accounted for 43.5% of the variance in the data. The components were found to be uncorrelated (component correlation −0.077) hence it was appropriate to use a Varimax rotation, treating the components as orthogonal. The rotated component loadings are shown in Table 2. A reliability analysis on the items belonging to the two components indicated that the 14 questions may be seen as 2 separate scales measuring distinct concepts, with 7 questions in each scale. Given that the 14 claims about NE were not designed to measure specific latent constructs the interpretation of the two scales is not entirely straightforward. However, it seems justifiable to observe that scale 1 contains items that are prescriptive and formulate views on how NE *should* be dealt with. These items pertain more to the potential harmful societal consequences of NE and involve protective measures. On the other hand, scale 2 contains items that are more proactionary and individualistic in nature. Hence, we label them ‘Societal/Protective (SP for short) and ‘Individual/Proactionary’ (IP for short) respectively. For the ‘Societal/Protective’ scale Cronbach’s alpha = 0.74; while for the ‘Individual/Proactionary’ scale Cronbach’s alpha = 0.73. Here, the term proactionary is appropriated from Fuller’s work [41] and is understood as an approach that is open to taking individual and societal risks in order to achieve a positive transformation of the human condition.

**Table 2:** Claims loading on societal protective and individual proactionary components

Rotated Component Matrix^a^		
Claim	Component	
	Societal / Protective	Individual / Proactionary
People should be content with their talents and abilities and not use artificial means to improve their performance	.708	
People’s achievements should come from their own effort and not from pills and devices	.722	
It is essential that public authorities oversee and control neuro-enhancement	.486	
Only people with a medical problem should have access to neuro-enhancement	.591	
People need to be protected from pressures to use neuro-enhancers	.571	
Neuro-enhancement should never be used on children	.449	
Neuro-enhancement will threaten social cohesion	.673	
If a neuro-enhancer is safe, it should be available as a consumer product	−.469	.567
Neuro-enhancement should be available to all those who might want it	−.553	.509
It is an expression of human nature to try to overcome the limitations of our body and mind		.578
I can imagine neuro-enhancement opening up fascinating new opportunities		.678
Some people will use neuro-enhancers to cope with increasing demands in life		.653
As life gets more pressured, neuro-enhancement may be the only way out		.501
Neuro-enhancement will increase competition between people		.564

^a^Varimax with Kaiser normalization, Coefficients below 0.4 suppressed

As described in the methods section, these 14 claims were presented to respondents in a randomised order following the two contrastive vignette experiments. Did the vignettes create a context effect in the responses to the 14 claims? To investigate this possibility regression models were fitted using the experimental manipulations as explanatory variables to predict scores on the two scales separately. We found that a few of the sixteen experimental conditions influenced the SP and IP scales. Controlling for other variables, respondents who were randomly allocated to an employment vignette with a high performing protagonist scored on average 0.082 points higher on SP than respondents who were allocated to a low-performing protagonist (*p* < 0.01). For the education context two experimental factors impacted significantly on the responses to the claims. Respondents allocated to a vignette with a male protagonist instead of a female one scored on average 0.074 points higher on SP (*p* < 0.05) and those assigned to a vignette describing a high performing protagonist vs those with a low performing one scored on average 0.112 points higher (*p* < 0.001).

For IP, a somewhat different pattern emerged. In the employment experiments, a high efficacy enhancer was associated with 0.097 points higher scores on IP compared to a low efficacy intervention (*p* < 0.01). The same was true for the education context, where a high efficacy NE increased average responses by 0.060 points compared to a low efficacy NE (*p* < 0.05). While the effects are statistically significant, the regression coefficients are marginal from a substantive point of view and the explained variation is equally small (maximum R^2^ = 0.002, that is 0.2%).

There is a moderate negative correlation between the two scales (Pearson’s *r* = −0.351, *p* < 0.001), showing, it might be thought, that the two scales capture opposing values. The idea of pairs of values in opposition is a basic feature of a contemporary theory of values, Schwartz places ten universal and core values in a circular structure depending on whether they are in opposition or complimentary. “One oppositional pair is openness to change versus conservatism. On this dimension, self-direction and stimulation values oppose security, conformity and tradition values” ([42]: 269). Hence we would expect those subscribing to individual proaction to be opposed to societal protection. To test this idea we recoded the original continuous variables into 3-point categorical variables using a tertile split. Table 3 shows that some people express agreement with both values simultaneously. It is notable that the proportion of those scoring low on SP or IP is very low, suggesting that the majority of respondents can identify, to some extent, with the two value orientations. 40.7% of respondents scored high on SP while having moderate scores on IP. 15% have high scores on both scales. In total, 60.8% of respondents have high SP scores, while 33.9% have high IP scores. This could be a sign of genuine ambivalence expressed as the parallel desire to protect societal values, while also pursuing individual enhancement.

**Table 3:** Cross tabulation of support for societal protective and individual proactionary values

	Individual/Proactionary value scale			
Societal/Protective Value Scale	0–3.33	3.34–6.66	6.67–10	Total
0–3.33	0.2%	0.3%	1.8%	2.3%
3.34–6.66	0.8%	19%	17%	36.8%
6.67–10	5.1%	40.7%	15%	60.8%
Total	6.1%	60%	33.9%	100%

Now we turn to the discussion of the contrastive vignette experiments. In a subsequent section, we will analyse how the different value orientations captured by the two scales are related to opinions expressed in the experiment.

#### Contrastive Vignette Experiments - Neuroenhancement in Education and Employment

We used ordinary least squares regression with the four experimental manipulations (gender of protagonist; pill or device; high or low performance and high or low efficacy) included in the models as dichotomous - dummy - variables.

We first consider the employment context and the effects of the experimental manipulations, looking at the entire dataset of 11 countries (*N* = 11,716). Table 4, model 1 shows the regression coefficients of the experimental manipulations. The protagonist’s gender and the method of NE intervention were not significant predictors of respondents’ judgments on whether they would use an enhancer. However, respondents were more likely to believe they would make the same decision to use the enhancer if the protagonist’s performance was low (β = −0.355; *p* < 0.001), and if the enhancer’s efficacy was high (β = 0.307, p < 0.001). In substantive terms, the effect of the experimental manipulations was low, yielding a change of around 0.3 units on the 11-point scale. The amount of variation explained by the model is 0.5%.

**Table 4:** Employment context regression coefficients

Employment Context	Model 1	Model 2	Model 3
R-squared	0.005	0.016	0.278
Constant term	3.191 (0.067)	3.705 (0.113)	2.193 (0.198)
Protagonist’s gender (Reference category Female)	0.044 (0.060)	0.052 (0.060)	0.017 (0.051)
Protagonist’s performance Good (reference category Failing)	−0.355*** (0.060)	−0.347*** (0.060)	−0.284*** (0.051)
NE-technology Pill (reference category: Device)	0.070 (0.060)	0.068 (0.060)	0.024 (0.051)
NE-efficacy High (reference category: Low)	0.307*** (0.060)	0.317*** (0.060)	0.233*** (0.051)
Respondent is female (reference category: Male)		−0.491*** (0.060)	−0.306*** (0.052)
Respondent age 25–34 (reference category 18–24)		0.041 (0.116)	0.016 (0.100)
Respondent age 35–44 (reference category 18–24)		−0.202 (0.113)	−0.172 (0.097)
Respondent age 45–54 (reference category 18–24)		−0.275* (0.113)	−0.204* (0.097)
Respondent age 55+ (reference category 18–24)		−0.552*** (0.104)	−0.283*** (0.089)
Respondent has a university degree (reference category: no degree)		−0.004 (0.068)	−0.101 (0.058)
Societal/Protective scale (11-point scale)			−0.444*** (0.016)
Individual/Proactionary scale (11-point scale)			0.759*** (0.017)

Standard errors in parentheses

* p < 0.05; ** p < 0.01; *** p < 0.001

Model 1 includes the 4 experimental manipulations represented as dummy variables

Model 2 includes Model 1 plus sociodemographic indicators

Model 3 includes Model 2 and SP and IP scales

In model 2, sociodemographic indicators are added. The effect of the experimental manipulations is only marginally affected, with the pattern of significant vs. non-significant predictors remaining unchanged. On average, female respondents gave lower scores than males on the response variable, by close to half a unit on the 11-point scale. Compared to the 18–24-year-old age group, those aged 45–54 and 55 or above gave increasingly lower scores on the response variable, while possession of a university degree had no effect on the dependent variable.

Finally, model 3 includes the scales, SP and IP, derived from the 14 claims about neuroenhancement described in the previous section. The most striking difference between models 2 and 3 is in the amount of variation explained by the regressions. Including the two scales increases the explanatory power of the model 17-fold, with the pattern of significant and non-significant variables staying the same. Controlling for the other variables in the model, each unit increase on the SP scale is associated with a 0.4-unit drop on the response variable, while each 1-unit increase on the IP scale corresponds to a 0.76-point increase on the dependent variable. This suggests that respondents who expressed concerns about the societal impacts of neuroenhancement and favoured a cautious, protective approach were less likely to endorse neuroenhancement in an employment context. On the other hand, respondents who showed agreement with the IP scale were more likely to be in favour of enhancement.

Turning to the education context, Table 5, model 1 shows the regression coefficients of the experimental manipulations on the key response variable. The variation explained by the models is similarly small - 0.6% - but as in the case of the employment context, some experimental manipulations exerted a significant effect on responses. The protagonist’s gender was insignificant. A pill versus a brain stimulation device led to lower average scores of around 0.3 units (*p* < 0.001). The protagonist’s high performance compared to low performance created lower willingness to use an enhancer (β = −0.274, p < 0.001), while higher efficacy NE interventions led to higher willingness to use (β = 0.336, p < 0.001) NE. Model 2 shows that female respondents expressed lower willingness to use a neuroenhancer than males (p < 0.001). With reference to the 18–24-year-old age group, those aged 35 or above gave lower scores on the response variable, with the 55+ age group being most different to the youngest (β = −0.803, p < 0.001). Having completed tertiary education made no difference to the responses.

**Table 5:** Education context regression coefficients

Education	Model 1	Model 2	Model 3
R-squared	0.006	0.018	0.270
Constant term	3.580 (0.069)	4.301 (0.117)	2.668 (0.205)
Protagonist’s gender (Reference category Female)	−0.039 (0.062)	−0.039 (0.062)	0.038 (0.053)
Protagonist’s performance Good (reference category Failing)	−0.274*** (0.053)	−0.286*** (0.062)	−0.206*** (0.053)
NE-technology Pill (reference category: Device)	−0.293*** (0.053)	−0.286*** (0.062)	−0.246*** (0.053)
NE-efficacy High (reference category: Low)	0.336*** (0.053)	0.341*** (0.062)	0.298*** (0.053)
Respondent is female (reference category: Male)		−0.489*** (0.062)	−0.301*** (0.053)
Respondent age 25–34 (reference category 18–24)		−0.199 (0.119)	−0.226* (0.103)
Respondent age 35–44 (reference category 18–24)		−0.478*** (0.117)	−0.446*** (0.100)
Respondent age 45–54 (reference category 18–24)		−0.433*** (0.116)	−0.363*** (0.100)
Respondent age 55+ (reference category 18–24)		−0.802*** (0.107)	−0.531*** (0.092)
Respondent has a university degree (reference category: no degree)		0.042 (0.070)	−0.054 (0.060)
Societal/Protective scale (11-point scale)			−0.450*** (0.017)
Individual/Proactionary scale (11-point scale)			0.764*** (0.017)

Standard errors in parentheses

* p < 0.05; ** p < 0.01; *** p < 0.001

Model 1 includes the 4 experimental manipulations represented as dummy variables

Model 2 includes Model 1 plus sociodemographic indicators

Model 3 includes Model 2 and SP and IP scales

As in the employment context, the amount of variation explained by model 3 is 15 times higher than that of model 2 (R^2^ = 0.27). Controlling for the other variables, each unit increase on the SP scale is associated with a 0.45-unit drop on the response variable, while each 1-unit increase on the IP scale corresponds to a 0.76-point increase.

It is important to note that the two contexts, education and employment are not directly comparable, because the depicted side effects (headache vs occasional insomnia), benefits (speed of learning vs memory and concentration) and the stakes involved in the two scenarios (exam performance vs job performance) were very different. Notwithstanding these crucial differences, we find that the protagonist’s level of performance and the enhancer’s efficacy were significant considerations in both contexts, which is in line with the pragmatic approach to NE identified by previous studies. Furthermore, in the education context pills were associated with lower willingness to use than brain stimulation. This finding is somewhat surprising, given that pharmaceuticals are much more familiar and we may reasonably assume that most respondents have had experiences with them, while electrical brain stimulation is a new and unfamiliar intervention. However, the negative effect of pills in the education context may be – at least partially – explained by long standing concerns and discussions about the use psychostimulant medications to address conditions such as attention deficit hyperactivity disorder [43]. A breakdown by country of Table 5 showing the regression coefficients (see Supplementary Material) shows that, with the exception of the two value scales, there are no consistent effects or remarkable differences between countries. We doubt whether the interpretation of the significant effects would justified.

## Conclusion

Large scale survey research assessing the public’s support for neuroenhancement in general showed that many people are in favour of the technology to improve the abilities of healthy people. By contrast, the experimental neuroethics literature indicates that people are sceptical about non-medical applications of neurotechnologies, identifying concerns about the safety of non-medical interventions, risks associated with coercion, inequity and authenticity. While therapeutic applications are generally acceptable, the enhancement of healthy people is not [22, 26, 27].

The current study draws on extensive public engagement activities to provide a bottom-up perspective on the ethics of enhancement and to combine this with the experimental and survey methodologies to investigate the public’s views about NE.

In contrastive vignette experiments we assessed how the gender of the protagonist, his or her level of performance, the efficacy of the enhancer and the mode of enhancement affects support for the use of NE in educational and employment contexts. Respondents were also asked the extent to which they agreed or disagreed with claims about NE that were frequently voiced in a series of public engagement exercises. The results of the experiments show that, as Sattler [24] had found, a pragmatic approach to decision taking is in evidence. We find that caution prevails when it comes to applications beyond medical use and the extent to which agreement to use NEs can be modified by such factors is small.

Looking at the aggregate data across eleven countries we find that there is no evidence that the use of NE by men and women is viewed differently. In the education context, there is more support for neuroenhancing electrical brain stimulation rather than pills. This finding may be related to a number of factors, including unfavourable representations of drug taking and illegal substances, or to widespread concerns and the high media visibility of debates about psychostimulant ADHD medications, as well as to the low levels of public trust in the pharmaceutical industry [44].

In both the education and employment contexts, when performance is already good respondents see little need for further enhancement. By contrast, NE is more acceptable when performance is low and an extra boost is needed to avoid failure. In both contexts, higher efficacy of NE is associated with greater willingness to use. There is a clear generational divide in willingness to support; the 18–24-year-old respondents show consistently higher agreement to use and as age increases so does agreement to use decline. When deciding whether to support NE or not respondents focus more on the anticipated benefits (efficacy) rather than the technology (pills or device).

The claims about NE show that over 70% agree that NE should never be used on children and that it needs oversight from public authorities. Yet, almost 70% agree that overcoming the limitations of mind and body is in human nature and more than 40% say that, if shown to be safe, NEs should be available as consumer products.

Looking at the responses to these claims with multivariate methods we find two distinct value orientations - the societal protective and the individual proactionary stances. These are strongly correlated with people’s views about the use of enhancement in employment and education.

The pursuit of the good life is an inextricable part of human nature, which is essentially underpinned by values. In social psychology, the concept of social values not only captures preferences and desires, but also expresses what should, and what should not, be deemed desirable. People feel committed to values, not because they have to, but because they want to [45]. We have seen the role of values very clearly in previous debates about science and technology, such as the public controversy over research on human embryonic stem cells. That debate was characterised by the competing demands of the values of ‘duty of care’ and the ‘sanctity of life’ [46]. In passing, with respect to enhancement it seems likely that the value of the ‘duty of care’ lies behind the almost universal support for therapeutic interventions. Traditionally, social values have been seen as stable, enduring and applying across all walks of life. On this perspective the two value orientations might be argued to reflect different attitudes towards the current and familiar state of affairs and to the risks associated with change.

The societal protective outlook views the status quo positively and as the right and proper state of affairs. People are rightly rewarded for hard work; they should be satisfied with their talents and abilities, medicines are only for people who are ill and people need to be protected from pressure to use NE. In contrast, the proactionary sees change and personal growth as a natural part of human motivation; human enhancement is a means to open up new opportunities for individual and social development and, if safe, enhancers should be consumer products. These value orientations result in different evaluations of the effects of NE on society. The proactionary view accepts that NE will increase competition between people and that it will be used to cope with increasing demands in life; the protective outlook fears that enhancement will threaten social cohesion.

The values of collectivism and individualism, once taken to be mutually exclusive, are now recognised as co-existing constructs both within cultures and individuals [47]. For many of our respondents NE is viewed through the two perspectives and simultaneously evokes ideas of concern and promise. Parens makes a similar point in a discussion of ethical frameworks that shape people’s responses to issues like enhancement technologies. He identifies a gratitude framework (be thankful for what we have) and a creativity framework (an obligation to transform ourselves). But crucially, he argues that “none of us who is thoughtful inhabits on only one of these frameworks” ([48]:38). This ambivalence suggests that there is a need for broad societal engagement with the questions raised by the prospect of enhancement in various spheres of life.

## Electronic supplementary material

ESM 1 (DOCX 16 kb)

## References

[1] Singh I, Kelleher KJ (2010). Neuroenhancement in young people: Proposal for research, policy, and clinical management. Ajob Neuroscience.

[2] Smith ME, Farah MJ (2011). Are prescription stimulants Bsmart pills^? The epidemiology and cognitive neuroscience of prescription stimulant use by normal healthy individuals. Psychological Bulletin.

[3] Kadosh RC, Levy N, O'Shea J, Shea N, Savulescu J (2012). The neuroethics of non-invasive brain stimulation. Current Biology.

[4] Kadosh RC (2013). Using transcranial electrical stimulation to enhance cognitive functions in the typical and atypical brain. Translational Neuroscience.

[5] Wexler, A. 2017. Who uses direct-to-consumer brain stimulation products, and why? *A study of home users of tDCS devices. Journal of Cognitive Enhancement.*: 1–21.

[6] Franke AG, Bagusat C, Dietz P, Hoffmann I, Simon P, Ulrich R, Lieb K (2013). Use of illicit and prescription drugs for cognitive or mood enhancement among surgeons. BMC Medicine.

[7] Maher, B. 2008. Poll results: Look who's doping. *Nature.*10.1038/452674a. 10.1038/452674a 18401370

[8] Singh I, Bard I, Jackson J (2014). Robust resilience and substantial interest: A survey of pharmacological cognitive enhancement among university students in the UK and Ireland. PLoS One.

[9] Sattler S, Jotterand F, Dubljević V (2016). Cognitive enhancement in Germany: Prevalence, attitudes,moral acceptability, terms, legal status, and the ethics debate. Cognitive enhancement: Ethical and policy implications in international perspectives.

[10] Ragan CI, Bard I, Singh I (2013). What should we do about student use of cognitive enhancers? An analysis of current evidence. Neuropharmacology.

[11] Roskies A (2002). Neuroethics for the new millenium. Neuron.

[12] ter Meulen, R., A. Mohammed, and Hall, W. (Eds.). 2017. *Rethinking cognitive enhancement*. Oxford University Press.

[13] Health Council of the Netherlands 2003. Human enhancement (ethics and health monitoring report 2003 no. 4). The Hague.

[14] MedicalAssociation B (2007). Boosting your brainpower: Ethical aspects of cognitive enhancements.

[15] Coenen, C., Schuijff, M., Smits, M., Klaassen, P., Hennen, L., Rader, M., Wolbring, G. 2009 Human enhancement: A report for the European Parliament science technology options assessment, Brussels.

[16] Comitato Nazionale per la Bioetica. 2013 Neuroscience and Pharmacological Cognitive Enhancement: Bioethical Aspects. http://bioetica.governo.it/media/171824/p106_2013_cognitive-enhancement_en.pdf Accessed 5 February 2018.

[17] Nuffield Councilon Bioethics (2013). Novel neurotechnologies; intervening in the brain.

[18] Presidential Commission for the study of Bioethical Issues 2015. Grey matters: Topics at the intersection of neuroscience, Ethics, and Society. http://bioethics.gov/sites/default/files/GrayMatter_V2_508.pdf Last Accessed 5 Feb 2018.

[19] Dijkstra AM, Schuijff M (2016). Public opinions about human enhancement can enhance the expertonly debate: A review study. Public Understanding of Science.

[20] Nadler R, Reiner PB (2011). Prototypes or pragmatics? The open question of public attitudes toward enhancement. AJOB Neuroscience.

[21] Gaskell G (2011). The 2010 Eurobarometer on the life sciences. Nature Biotechnology.

[22] Schelle, K.J., N. Faulmüller, L. Caviola, and M. Hewstone. 2014. Attitudes toward pharmacological cognitive enhancement—A review. *Frontiers in Systems Neuroscience.*10.3389/fnsys.2014.00053. 10.3389/fnsys.2014.00053 PMC4029025 24860438

[23] Sattler, S., G. Mehlkop, P. Graeff, and C. Sauer. 2014. Evaluating the drivers of and obstacles to the willingness to use cognitive enhancement drugs: The influence of drug characteristics, social environment, and personal characteristics. *Substance Abuse Treatment, Prevention, and Policy.*10.1186/1747-597X-9-8. 10.1186/1747-597X-9-8 PMC3928621 24484640

[24] Sattler S, Forlini C, Racine É, Sauer C (2013). Impact of contextual factors and substance characteristics on perspectives toward cognitive enhancement. PLoS One.

[25] Sattler S, Sauer C, Mehlkop G, Graeff P (2013). The rationale for consuming cognitive enhancement drugs in university students and teachers. PLoS One.

[26] Fitz N, Nadler R, Manogaran P, Chong EW, Reiner PB (2014). Public attitudes toward cognitive enhancement. Neuroethics.

[27] Cabrera LY, Fitz NS, Reiner PB (2015). Empirical support for the moral salience of the therapy-enhancement distinction in the debate over cognitive, affective and social enhancement. Neuroethics.

[28] Forlini C, Hall W (2016). The is and ought of the ethics of Neuroenhancement: Mind the gap. Frontiers in Psychology.

[29] Zwart, H., J. Brenninkmeijer, P. Eduard, L. Krabbenborg, S. Laursen, G. Revuelta, and W. Toonders. 2017. Reflection as a deliberative and distributed practice: Assessing neuroenhancement technologies via mutual learning exercises (MLEs). *NanoEthics*. 10.1007/s11569-017-0287-4. 10.1007/s11569-017-0287-4 PMC5554482 28845202

[30] Burstin, Kenneth, Eugene B. Doughtie, and Avi Raphaeli. 1980. Contrastive vignette technique: An indirect methodology designed to address reactive social attitude measurement. *Journal of Applied Social Psychology.*10.1111/j.1559-1816.1980.tb00699.x.

[31] Bostrom N, Sandberg A (2009). Cognitive enhancement: Methods, ethics, regulatory challenges. Science and Engineering Ethics.

[32] Farah MJ, Illes J, Cook-Deegan R, Gardner H, Kandel E, King P (2004). Neurocognitive enhancement: What can we do and what should we do?. Nature Reviews Neuroscience.

[33] Cohen IG (2013). What (if anything) is wrong with human enhancement; what (is anything) is right with it. Tulsa L. Rev..

[34] Garasic MD, Lavazza A (2016). Moral and social reasons to acknowledge the use of cognitive enhancers in competitiveselective contexts. BMC Medical Ethics.

[35] Kahneman D, Tversky A (1979). Prospect theory: Ananalysis of decision under risk. Econometrica: Journal of the Econometric Society.

[36] Jwa A (2015). Early adopters of the magical thinking cap: A study on do-it-yourself (DIY) transcranial direct current stimulation (tDCS) user community. Journal of Law and the Biosciences.

[37] Maslen H, Douglas T, Kadosh RC, Levy N, Savulescu J (2015). Do-it-yourself brain stimulation: A regulatory model. Journal of Medical Ethics.

[38] Nichols AL, Maner JK (2008). The good-subject effect: Investigating participant demand characteristics. The Journal of General Psychology.

[39] Abelson, R.P. 1976. Script processing in attitude formation and decision making. In (1976). Cognition and social behavior, ed. John S. Carroll and John W. Payne. Oxford: Lawrence Erlbaum.

[40] Specker J, Schermer MH, Reiner PB (2017). Public attitudes towards moral enhancement. Evidence that means matter morally. Neuroethics.

[41] Fuller, S., Lipinska, V. 2014. The Proactionary imperative: A Foundation for Transhumanism. Springer.

[42] Schwartz, S.H. 2003. A proposal for measuring value orientations across nations. *Questionnaire Package of the European Social Survey*: 259–290.

[43] Bergey, M. R., & Conrad, P. 2018. The Rise and Transformation of ADHD in the United States. Global Perspectives on ADHD: Social Dimensions of Diagnosis and Treatment in Sixteen Countries, 9.

[44] Goldacre B (2012). Bad pharma: How medicine is broken, and how we can fix it.

[45] Tsirogianni S, Gaskell G (2011). The role of plurality and context in social values. Journal for the Theory of Social Behaviour.

[46] Gaskell G, Stares S, Pottage A (2012). How Europe's ethical divide looms over biotech law and patents. Nature Biotechnology.

[47] Braithwaite V, Makkai T, Pittelkow Y (1996). Inglehart's materialism-Postmaterialism concept: Clarifying the dimensionality debate through Rokeach's model of social values. Journal of Applied Social Psychology.

[48] Parens E (2005). Authenticity and ambivalence: Toward understanding the enhancement debate. Hastings Center Report.

[49] ESS Round 2: European Social Survey Round 2 Data. 2004. Data file edition 3.5. NSD-Norwegian Centre for Research Data, Norway–Data Archive and distributor of ESS data for ESS ERIC.

