# Supplementary material for: Bottom Up Ethics - Neuroenhancement in Education and Employment
Source: Neuroethics. 2018 May 1;11(3):309–22. doi: 10.1007/s12152-018-9366-7 (PMC6132847; doi:10.1007/s12152-018-9366-7)
Supplement: Supplementary file 1 — (DOCX 16 kb) [file 12152_2018_9366_MOESM1_ESM.docx]

*Supplementary Table 1. Education and employment context regression coefficients of response variable “In the protagonist’s shoes, would you do the same?” for each participating country. Models include the 4 experimental manipulations represented as dummy variables, sociodemographic indicators, and SP and IP scales. * p<0.05; ** p<0.01; *** p<0.001*

| **EDUCATION context** | | | | | | | | | | | |  |
| --- | --- | --- | --- | --- | --- | --- | --- | --- | --- | --- | --- | --- |
|  | **AT** | **DK** | **DE** | **H** | **IS** | **IT** | **NL** | **PT** | **ES** | **UK** | **US** | |
| **Constant** | 2.460 | 4.393 | 3.801 | 3.985 | 2.302 | 2.135 | 3.570 | 1.251 | 1.606 | 4.018 | 1.312 | |
| **Male vs Female protagonist** |  |  |  |  |  |  |  |  |  |  |  | |
| **Pill vs device** | -0.775*** | -0.448** | -0.869*** |  |  |  |  | 0.526** |  | -0.489** |  | |
| **High vs low efficacy** | 0.609*** | 0.441** |  |  | 0.435* | 0.348* | 0.376* |  |  |  | 0.644*** | |
| **Good vs failing performance** |  |  | -0.373* |  | -0.484** |  |  |  | 0.368* | -0.430** |  | |
| **Female vs male respondent** |  |  |  |  |  |  | -0.410* |  |  | -0.511** | -0.396* | |
| **25-34 vs 18-24** |  |  |  |  |  |  |  |  |  |  |  | |
| **35-44 vs 18-24** |  |  |  |  |  | -0.752* |  |  |  | -0.863** | -0.780* | |
| **45-54 vs 18-24** |  |  |  |  |  |  |  |  |  |  | -0.974** | |
| **55+ vs 18-24** |  | -0.926*** |  |  |  | -0.974*** |  |  | -0.887** | -0.996*** | -1.257*** | |
| **Uni degree vs no degree** |  |  | -0.477* |  |  | 0.457* |  |  |  |  |  | |
| **Societal/Protective** | -0.385*** | -0.584*** | -0.470*** | -0.540*** | -0.462*** | -0.474*** | -0.541*** | -0.453*** | -0.485*** | -0.579*** | -0.326*** | |
| **Individual/Proactionary** | 0.782*** | 0.678*** | 0.782*** | 0.656*** | 0.737*** | 0.790*** | 0.747*** | 0.721*** | 0.890*** | 0.866*** | 0.931*** | |
| **Explanatory power (R^2^)** | 0.270 | 0.298 | 0.299 | 0.253 | 0.260 | 0.343 | 0.293 | 0.193 | 0.311 | 0.394 | 0.325 | |
| **EMPLOYMENT context** | | | | | | | | | | | |  |
| **Constant** | 2.619 | 4.011 | 2.831 | 3.057 | 0.44 | 2.824 | 4.04 | 0.945 | 1.109 | 3.008 | 1.518 | |
| **Male vs Female protagonist** |  |  |  |  |  | -0.323* |  |  |  |  |  | |
| **Pill vs device** | -0.660*** |  |  |  |  | 0.822*** |  | 0.422* |  |  |  | |
| **High vs low efficacy** |  | 0.326* | 0.447** |  | 0.389* |  | 0.407** |  |  | 0.350* | 0.394* | |
| **Good vs failing performance** |  |  |  |  | -0.445* |  | -0.408*** | -0.395* |  |  | -0.504** | |
| **Female vs male respondent** |  | -0.640*** | -0.366* |  |  |  | -0.503*** |  | -0.440** | -0.454** | -0.414* | |
| **25-34 vs 18-24** |  |  |  |  |  |  |  |  |  |  |  | |
| **35-44 vs 18-24** |  | -0.953*** |  |  |  |  |  |  |  |  |  | |
| **45-54 vs 18-24** |  | -0.972*** |  |  |  |  |  |  |  |  |  | |
| **55+ vs 18-24** |  | -1.223*** |  |  |  | -0.718* |  |  |  |  | -1.094*** | |
| **Uni degree vs no degree** |  |  |  |  |  |  |  |  |  | -0.558** |  | |
| **Societal/Protective** | -0.418*** | -0.517*** | -0.530*** | -0.481*** | -0.316*** | -0.546*** | -0.570*** | -0.459*** | -0.504*** | -0.474*** | -0.321*** | |
| **Individual/Proactionary** | 0.723*** | 0.676*** | 0.726*** | 0.618*** | 0.733*** | 0.742*** | 0.640*** | 0.827*** | -0.914*** | 0.746*** | 0.922*** | |
| **Explanatory power (R^2^)** | 0.264 | 0.333 | 0.308 | 0.232 | 0.233 | 0.350 | 0.290 | 0.240 | 0.344 | 0.308 | 0.319 | |
